# Comprehensive profiling of the human viral exposome in households containing an at‐risk child with mitochondrial disease during the 2020–2021 COVID‐19 pandemic

**DOI:** 10.1002/ctm2.1100

**Published:** 2022-11-06

**Authors:** Eliza M. Gordon‐Lipkin, Christopher S. Marcum, Shannon Kruk, Elizabeth Thompson, Sophie E. M. Kelly, Heather Kalish, Lorenza Bellusci, Surender Khurana, Kaitlyn Sadtler, Peter J. McGuire

**Affiliations:** ^1^ Metabolism, Infection and Immunity Section National Human Genome Research Institute National Institutes of Health Bethesda Maryland USA; ^2^ Data Science Policy National Institute of Allergy and Infectious Diseases National Institutes of Health Bethesda Maryland USA; ^3^ Trans‐NIH Shared Resource on Biomedical Engineering and Physical Science National Institute of Biomedical Imaging and Bioengineering National Institutes of Health Bethesda Maryland USA; ^4^ Division of Viral Products Center for Biologics Evaluation and Research Food and Drug Administration (FDA) Silver Spring Maryland USA; ^5^ Section on Immunoengineering National Institute of Biomedical Imaging and Bioengineering National Institutes of Health Bethesda Maryland USA

**Keywords:** COVID‐19, infection, mitochondrial disease, viral exposome, VirScan

## Abstract

**Background:**

Viral infection is a major cause of morbidity in children with mitochondrial disease (MtD). As a result, families with children with MtD are highly adherent to risk mitigation behaviours (RMBs) advised by the Centers for Disease Control and Prevention during the COVID‐19 pandemic that can modulate infection risk.

**Methods:**

Deep serologic phenotyping of viral infections was performed via home‐based sampling by combining SARS‐CoV‐2 serologic testing and phage display immunoprecipitation and sequencing. Samples were collected approximately 1 year apart (October 2020 to April 2021 and October 2021 to March 2022) on households containing a child with MtD.

**Results:**

In contrast to our first collection in 2020–2021, SARS‐CoV‐2 antibody profiles for all participants in 2021–2022 were marked by greater isotype diversity and the appearance of neutralizing antibodies. Besides SARS‐CoV‐2, households (*N* = 15) were exposed to >38 different respiratory and gastrointestinal viruses during the study, averaging five viral infections per child with MtD. Regarding clinical outcomes, children with MtD (*N* = 17) experienced 34 episodes of illness resulting in 6 hospitalizations, with some children experiencing multiple episodes. Neurologic events following illness were recorded in five patients. Infections were identified via clinical testing in only seven cases. Viral exposome profiles were consistent with clinical testing and even identified infections not captured by clinical testing.

**Conclusions:**

Despite reported adherence to RMBs during the COVID‐19 pandemic by families with a child with MtD, viral infection was pervasive. Not all infections resulted in illness in the child with MtD, suggesting that some were subclinical or asymptomatic. However, selected children with MtD did experience neurologic events. Our studies emphasize that viral infections are inexorable, emphasizing the need for further understanding of host‐pathogen interactions through broad serologic surveillance.

## Graphical abstract

In families with a child with MtD:

- Home‐based testing of the viral exposome was conducted.
- Despite adherence to risk mitigation behaviours, viral infection was pervasive.
- Children with MtD experienced neurologic events surrounding episodes of viral infection.
- Vaccination results in more robust and diverse immune responses to SARS‐CoV‐2.

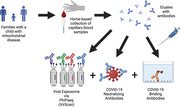

## 1 BACKGROUND

Viral infections are exceedingly prevalent, being more common amongst young children, due to incomplete immunity.^1^, ^2^, ^3^ In the United States, approximately 25 million children are treated for upper respiratory tract infections in outpatient clinical settings annually, and acute respiratory infections cause considerable morbidity and mortality worldwide.^4^ Concerning GI infections, acute diarrhoeal episodes in children occur frequently in daycare and hospital settings, with rotavirus being the usual cause.^5^, ^6^ Children with neurodevelopmental disabilities, such as mitochondrial disease, are at increased risk for significant morbidity and mortality due to viral infections.^7^

Deleterious variants in nDNA and mtDNA genes involved in mitochondrial function lead to disorders of oxidative phosphorylation, collectively known as mitochondrial diseases (MtDs). MtDs are the most common inborn errors of metabolism with a minimum prevalence of ≈1 in 5000.^8^ The phenotype of MtD is multisystemic, involving organ systems with large energy requirements (e.g. central nervous system [CNS]). Infection is a major cause of morbidity in children with MtD and can precipitate acidosis and organ dysfunction with a rapidly fatal course.^9^ More than 80% of children with MtD may experience recurrent or severe infections,^10^ and sepsis and pneumonia are common causes of death.^11^ Following respiratory viral infections, up to half of patients may experience life‐threatening or neurologic sequelae.^12^ As such, viral infections pose a palpable and unpredictable threat to children with MtD, their caregivers and medical providers.

Public health messaging via the Centers for Disease Control (CDC) during the COVID‐19 pandemic advised risk mitigation behaviours (RMBs) to lessen transmission risk. RMBs are non‐pharmaceutical interventions used to mitigate the effects of COVID‐19 (e.g. handwashing, mask wearing and physical distancing). These behaviours were found to not only impact SARS‐CoV‐2, but also other viral transmission risks.^13^, ^14^ However, the home remains a substantial hub for infection, with secondary attack rates that vary depending on the infectious agent, comorbidities and the vaccination status of the household members.^15^, ^16^, ^17^ As such, we hypothesized that viral infection is pervasive, and households would demonstrate serologic markers of viral exposure, potentially endangering the child with MtD. To understand the breadth of viral infections (i.e. viral exposome) and risk posed by the home environment, we conducted a household‐based serologic study and monitored clinical outcomes in focused cohort of children with MtD at risk for adverse outcomes due to viral infection. The study was conducted through 2020 and 2021 of the COVID‐19 pandemic and is the first report of deep phenotyping of viral infections and clinical correlates in this vulnerable population of patients.

## 2 METHODS

### 2.1 Standard protocol approvals, registrations and patient consents

The cohort of patients and their families (*N* = 20 families) were self‐selected participants in a natural history study of infection in children with MtD (ClinicalTrials.gov identifier: NCT04419870). This study was approved by the National Institutes of Health Institutional Review Board. Consent was obtained prior to study enrolment.

### 2.2 Subject characteristics

The characteristics of the children with MtD (*N* = 22) are shown in Table 1. The mean age was 8.8 (±4.4 standard deviation [SD]) years of age, with similar proportions of male (*N* = 11) and female (*N* = 11) subjects (Table 2). MtD subjects had molecular confirmation and included the following phenotypes: Leigh syndrome (*N* = 11), Leigh‐like syndrome (*N* = 4), MERFF (*N* = 1), MELAS (*N* = 2) and MtD not otherwise specified (NOS, *N* = 4) (Table S1). Clinical records, including neuroimaging records, were obtained by the NIH team and reviewed by a neurologist with expertise in MtD (E.G.L.) to determine MtD phenotype. CNS findings, previously identified on MRI, were found in 82% of children with MtD. MtD disease severity was measured using Domain 1 (parent/caregiver interview) of the International Paediatric Mitochondrial Disease Scale (IPMDS‐D1).^18^ The median IPMDS‐D1 score was 17 (range 2–48), placing most patients in the moderate‐to‐severe range of disease.

**TABLE 1:** Seropositivity profile in participants with anti‐SARS‐CoV‐2 binding antibodies

	2020			2021						
	Fam	MtD	Fam versus MtD	Fam	MtD	Fam versus MtD	2020–2021 Fam		2020–2021 MtD	
Nucleocapsid	Number (%)		*p*‐Value	Number (%)		*p*‐Value	Change (%)	*p*‐Value	Change (%)	*p*‐Value
*IgG^+^*	6/61 (10)	2/22 (9.1)	1.00	19/41 (46)	7/17 (41)	.78	36	**<.00001**	32	**.02**
Spike										
*IgG^+^ IgM^+^ IgA^+^*	8/61 (13)	1/22 (4.5)	.43	32/41 (78)	9/17 (53)	.07	65	**<.00001**	49	**.0009**
*IgG^+^ IgM^+^*	2/61 (3.2)	0/22 (.0)	1.00	1/41 (2.4)	0/17 (.0)	1.00	−.8	1.00	.0	1.00
*IgG^+^ IgA^+^*	3/61 (4.9)	0/22 (.0)	.56	4/41 (9.8)	1/17 (5.9)	1.00	4.9	.43	5.9	.44
*IgM^+^ IgA^+^*	1/61 (1.6)	0/22 (.0)	1.00	0/41 (.0)	0/17 (.0)	1.00	−1.6	1.00	.0	1.00
*IgG^+^*	1/61 (1.6)	1/22 (4.5)	.46	2/41 (4.8)	0/17 (.0)	1.00	3.2	1.00	−4.5	1.00
*IgM^+^*	11/61 (18)	9/22 (41)	**.04**	3/41 (7.3)	4/17 (24)	.18	−11	.15	−17	.32
*IgA^+^*	1/61 (1.6)	0/22 (.0)	1.00	0/41 (.0)	0/17 (.0)	1.00	−1.6	1.00	.0	1.00
*Any IgG^+^*	14/61 (23)	2/22 (9.1)	.21	38/41 (93)	10/17 (59)	**.004**	70	**<.00001**	50	**.001**
*Any IgM^+^*	22/61 (36)	9/22 (41)	.61	35/41 (85)	13/17 (76)	.46	49	**<.00001**	35	**.04**
RBD										
*IgG^+^ IgM^+^ IgA^+^*	7/61 (11)	0/22 (.0)	.18	29/41 (71)	8/17 (47)	.13	60	**<.00001**	47	**.0004**
*IgG^+^ IgM^+^*	29/61 (48)	10/22 (45)	1.00	9/41 (22)	4/17 (24)	1.00	−26	**.01**	−21	.19
*IgG^+^ IgA^+^*	0/61 (.0)	0/22 (.0)	1.00	2/41 (4.8)	1/17 (5.9)	1.00	4.8	.15	5.9	.44
*IgM^+^ IgA^+^*	1/61 (1.6)	0/22 (.0)	1.00	0/41 (.0)	0/17 (.0)	1.00	−1.6	1.00	.0	1.00
*IgG^+^*	7/61 (11)	2/22 (9.1)	1.00	3/41 (7.3)	0/17 (.0)	.55	−3.7	.74	−9.1	.49
*IgM^+^*	7/61 (11)	8/22 (36)	**.02**	0/41 (.0)	1/17 (5.9)	.29	−11	**.04**	−31	**.03**
*IgA^+^*	0/61 (.0)	0/22 (.0)	1.00	0/41 (.0)	0/17 (.0)	1.00	.0	1.00	.0	1.00
*Any IgG^+^*	43/61 (70)	12/22 (55)	.2	41/41 (100)	13/17 (76)	**.006**	30	**<.00001**	21	.19
*Any IgM^+^*	44/61 (72)	18/22 (82)	.57	36/41 (88)	13/17 (76)	.43	16	.82	−6.0	.71

*Note*: Binding antibodies indicate pathogen exposure. Serum was extracted from the at‐home collection apparatus and tested for antibodies by ELISA against various components of SARS‐CoV‐2. Each row signifies the antibody or combination of antibodies detected for each type of protein. Only testing for nucleocapsid IgG binding antibody was available at the time of the study. The prefix ‘Any’ (e.g. Any IgM^+^) signifies the presence of said isotype regardless of whether it is found in combination with other isotypes. ‘Change’ indicates the change in positivity from the previous time period: 2020 = 2020–2021 winter season; 2021 = 2021–2022 winter season. Fisher's exact test with *p* < .05 in bold.

Abbreviations: Fam, family members; IgA, immunoglobulin A; IgG, immunoglobulin G; IgM, immunoglobulin M; MtD, mitochondrial disease; RBD, receptor‐binding domain; Spike, spike protein.

**TABLE 2:** Top 10 viruses detect by the Antiviral Antibody Response Deconvolution Algorithm (AVARDA)

	Fam	MtD		
Virus	Number (%)		*p*‐Value	Shared
*Enterovirus B*	9/39 (23)	9/17 (53)	.06	5/17 (29)
*Rhinovirus A*	12/39 (31)	9/17 (53)	.14	3/17 (18)
*Enterovirus C*	3/39 (7.7)	8/17 (47)	**.002**	4/17 (24)
*Rhinovirus B*	7/39 (18)	5/17 (29)	.48	1/17 (5.9)
*Enterovirus A*	4/39 (10)	4/17 (24)	.23	2/17 (12)
*Influenza B virus*	6/39 (15)	4/17 (24)	.47	1/17 (5.9)
*Respiratory syncytial virus*	6/39 (15)	4/17 (24)	.47	3/17 (18)
*Enterovirus D*	3/39 (7.7)	3/17 (18)	.35	1/17 (5.9)
*Human mastadenovirus D*	0/39 (.0)	3/17 (18)	**.02**	3/17 (18)
*SARS related coronavirus*	3/39 (7.7)	3/17 (18)	.35	1/17 (5.9)

*Note*: Capillary blood samples were eluted from the collection apparatus (Neoteryx) and used to detect antiviral antibodies via phage display immunoprecipitation sequencing (VirScan). Viral infections were called via a deconvolution algorithm (AVARDA). Virus exposures for the period of study were determined by comparing antiviral antibody profiles between the first and second collections. Fisher's exact test with *p* < .05 in bold.

Abbreviations: Fam, family members; MtD, mitochondrial disease.

### 2.3 Sample collection

At‐home collection of capillary blood samples (30 μl) was performed using a Mitra Specimen Collection Kit (Neoteryx, Torrance, CA) and a return shipping label. Upon receipt, microsamplers were stored dry at −80°C until elution and analysis. Corresponding clinical information was collected by telephone and encrypted online questionnaires.

### 2.4 Serologic assays

The determination of SARS‐CoV‐2 antibodies from microsamples has been previously published by our co‐authors.^19^ Briefly, whole blood samples were loaded onto 30 μl Neoteryx Mitra Microsampling device tips, dried and stored in 500 μl Eppendorf tubes at −80°C. Microsampler tips were eluted in 500 μl of 1 × PBS (Gibco) + 1%BSA + .5%Tween (Sigma‐Aldrich, St. Louis, MO) at 4°C at 3000 rpm. The eluate was stored at −80°C until use. Nucleocapsid, spike protein and receptor‐binding domain (RBD) binding antibodies were analysed using an enzyme‐linked immunosorbent assay.^19^, ^20^, ^21^ Longitudinal quality control and assay stability was ensured by the inclusion of controls on each plate (Recombinant anti‐SARS‐CoV‐2 RBD, GenScript, and anti‐SARS‐CoV‐2 Nucleocapsid, ThermoFisher Scientific, Waltham, MA). Seropositivity cut points were defined previously with thresholds based on the mean optical density (absorbance) +3 SDs.

### 2.5 SARS‐CoV‐2 spike‐expressing pseudovirions for viral neutralization assays

Human samples were evaluated in a qualified SARS‐CoV‐2 pseudovirion neutralization assay (PsVNA) using SARS‐CoV‐2 WA1/2020 strain (CDC reference strain) and variants. SARS‐CoV‐2 neutralizing activity measured by PsVNA correlates with PRNT (plaque reduction neutralization test with authentic SARS‐CoV‐2) in the previous studies.^22^, ^23^, ^24^ PsVNA were produced as previously described.^24^ Briefly, human codon‐optimized cDNA encoding SARS‐CoV‐2 spike glycoprotein of the WA1/2020 and variants were synthesized by GenScript and cloned into eukaryotic cell expression vector pcDNA 3.1 between the *BamH*I and *Xho*I sites. PsVNA were produced by co‐transfection Lenti‐X 293T cells with psPAX2(gag/pol), pTrip‐LUC lentiviral vector and pcDNA 3.1 SARS‐CoV‐2‐spike‐deltaC19, using Lipofectamine 3000. The supernatants were harvested at 48 h post‐transfection and filtered through .45 μm membranes and titrated using 293T‐ACE2‐TMPRSS2 cells (HEK 293T cells that express ACE2 and TMPRSS2 proteins).

### 2.6 SARS‐CoV‐2 neutralization assays

Neutralization assays were performed as previously described.^22^, ^23^, ^25^, ^26^, ^27^ For the neutralization assay, 50 μl of SARS‐CoV‐2 S pseudovirions (counting ∼200000 relative light units) were pre‐incubated with an equal volume of medium containing serial dilutions of samples at room temperature for 1 h. Then 50 μl of virus–antibody mixtures were added to 293T‐ACE2‐TMPRSS2 cells (10^4^ cells/50 μl) in a 96‐well plate. The input viruses with all SARS‐CoV‐2 strains used in the current study were the same (2 × 10^5^ relative light units/50 μl/well). After a 3 h incubation, fresh medium was added to the wells. Cells were lysed 24 h later, and luciferase activity was measured using ONE‐Glo luciferase assay system (Promega, Cat# E6130). The assay of each sample was performed in duplicate, and the 50% neutralization titre was calculated using Prism 9 (GraphPad Software). Controls included cells only, virus without any antibody and positive sera.

### 2.7 Phage display immunoprecipitation sequencing (PhIP‐Seq, VirScan)

Neoteryx eluates (extraction described above in Section 2.4) were stored at −80°C until use. VirScan services were provided by CDI Labs (Baltimore, MtD). In brief, library cloning, sample screening, PCR and peptide read count data curation are performed as described in Mohan et al.^28^ The VirScan library comprises over 110000 56‐mer overlapping peptides encapsulating the human viral proteome. Briefly, screens are performed in 1 ml PBS, pH 7.4 containing phage library (∼10^11^ pfu) and .2 μl of eluate. A set of eight independent buffer alone samples to which eluate is not added is included as negative control (beads only). The mixture is rotated overnight at 4°C. The next day, 40 μl of a 1:1 Protein A/G‐coated magnetic bead slurry is added and rotated for an additional 4 h at 4°C. The beads are then washed three times with TBS, pH 7.4 containing .1% NP‐40 and resuspended in 20 μl of a Herculase II Fusion Polymerase PCR1 master mix (Agilent Genomics, Santa Clara, CA). After 20 cycles of PCR, sample‐specific barcoding and the Illumina P5/P7 (Illumina, San Diego, CA) adapters are then incorporated during a subsequent PCR2 reaction. PCR2 amplicons are pooled and sequenced using an Illumina NextSeq to obtain single‐end 50 nucleotide reads. Demultiplexed reads are aligned to the human peptide library using exact matching. The R software package edgeR (www.r‐project.org) then compares the reads in each sample against the buffer alone (beads only) ‘mock’ immunoprecipitations using a negative binomial model. The software returns both a test statistic and fold‐change value for each peptide. Enriched peptides (hits) require counts, *p*‐values and fold changes of at least 15, .001 and 5, respectively.

### 2.8 AntiViral Antibody Response Deconvolution Algorithm (AVARDA)

To account for disproportionate representation of viruses in the library or for antibody cross‐reactivity among sequences shared by related viruses, viral exposure was determined by the AntiViral Antibody Response Deconvolution Algorithm (AVARDA).^29^ Briefly, AVARDA provides a probabilistic calculation of infection by alignment of all library peptides to each other and to all human viruses. Viral hits for individuals were called where either a new virus occurred with the second collection, or differential analysis via AVARDA revealed an increased enrichment in viral peptides.

### 2.9 Statistics

Statistical analyses were performed using Microsoft Excel (Microsoft, Redmond, WA) and Graph Pad Prism (San Diego, CA). Data are presented as continuous variables, counts, percentages and means ± SD. Fisher's exact test, Student's *t* test and two‐way ANOVA with multiple comparisons were used as appropriate. *p* < .05 was statistically significant.

### 2.10 Data availability

Data not provided in the article because of space limitations may be shared (anonymized) at the request of any qualified investigator for purposes of replicating procedures and results.

## 3 RESULTS

### 3.1 SARS‐CoV‐2 infections

Beginning in 2020, the COVID‐19 pandemic, caused by SARS‐CoV‐2, has led to serious illness in the United States and around the world.^30^ Early in the pandemic, before vaccines became widely available to the public, individuals with pre‐existing medical conditions such as children with MtD were particularly at risk for adverse outcomes.^31^ To understand the impact of COVID‐19 on a group of medically vulnerable children, we followed a focused cohort of children with MtD with risk factors for adverse outcomes following viral infection during the pandemic. All participants had molecular confirmation of their disease (*N* = 22, Table S1). Besides the hazards posed by having MtD, comorbidities and other risk factors for adverse outcomes following infection for children with MtD were also documented (Figure S1). All children had one or more risk factors. One of the most common comorbidities for adverse outcomes during viral infections was seizures (43%).^32^ Other risk factors of concern included being immunocompromised (23%), using immunomodulatory therapies (13%) and requiring respiratory support (18%).

**FIGURE 1:**
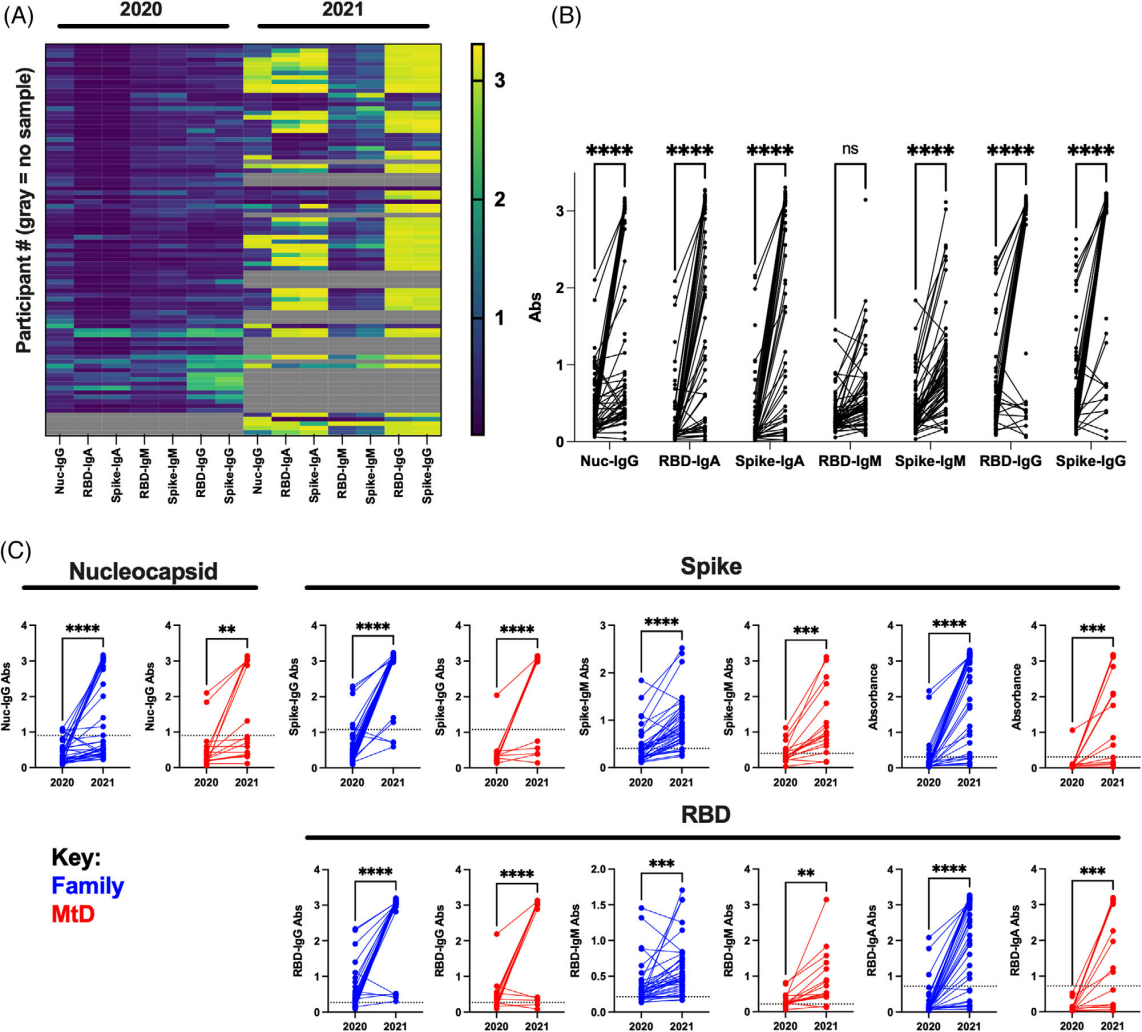
SARS‐CoV‐2 binding antibodies in family members and children with mitochondrial disease (MtD). Capillary blood samples were collected using a specialized sampling apparatus approximately 1 year apart. Samples were eluted from the sampling apparatus, and binding antibodies for viral proteins were determined by ELISA. Absorbance cut‐off values for positivity were determined previously. Binding antibodies define SARS‐CoV‐2 exposure. (A) Heat map showing binding antibody prevalence in participants. Yellow colour indicates higher absorbance levels. Grey boxes indicate absent data for that timepoint. Nuc, nucleocapsid; S, full spike ectodomain; RBD, spike receptor–binding domain. (B) Change in binding antibody absorbances from 2020–2021. (C) Breakdown of participants for nucleocapsid, spike and RBD binding antibodies 2020–2021. Two‐way ANOVA with mixed‐effects analysis and Student's *t* test were used as appropriate. NS, not significant. **p < .01, ***p < .001, ****p < .0001.

To obtain samples for our serologic study, capillary blood was collected from household members utilizing a microsampling apparatus. As SARS‐CoV‐2 was an emerging infection, we performed traditional serologic profiling developed by our co‐authors. Following extraction, we performed serologic studies for IgM, IgG and IgA binding antibodies against viral nucleocapsid, spike protein and RBD. These binding antibodies define SARS‐CoV‐2 exposure. IgM binding antibodies indicated recent exposure, whereas IgG and IgA binding antibodies indicated previous exposure. At our initial collection (households *N* = 20, participants *N* = 83), we found that 76% of participants were seropositive for at least one of the SARS‐CoV‐2‐binding antibodies (Figure 1A). Following our second collection (*N* = 15 households, *N* = 58 participants), the number of seropositive participants had risen to 92%. For subjects where samples were available for both timepoints (*N* = 41 family members, *N* = 17 MtD), binding antibody absorbances significantly increased for nucleocapsid, spike and RBD IgG, as well as spike and RBD IgA (Figure 1B, *p* < .0001). To understand the status of our participants, we next examined the change in binding antibody absorbance for children with MtD and family members. For both groups, nucleocapsid IgG, spike IgG, IgM and IgA, and RBD IgG, IgM and IgA increased (Figure 1C, *p* < .01 for all).

With serologic evidence of a more robust immune response to SARS‐CoV‐2 following our second collection, we next summarized the antibody profiles (Table 1). The breakdown of binding antibody profiles for each type of viral protein was presented in rows. The prefix ‘Any’ (e.g. Any IgM^+^) indicated the presence of said antibody regardless of whether it was found in combination with other isotypes. For nucleocapsid binding antibodies, the frequency of positive cases in family members (36% increase, *p* < .00001) and children with MtD (32% increase, *p* = .02) had risen similarly over the period of study. As nucleocapsid binding antibodies are not produced following vaccination, these represent natural infections. Next, we examined the frequency of spike and RBD binding antibody seropositivity. In 2020–2021, spike protein‐binding antibody profiles were mostly similar for family members and children with MtD, except for IgM. A spike‐binding antibody profile of entirely spike IgM^+^ was more frequent in children with MtD (18% vs. 41%, *p* = .04). RBD‐binding antibody profiles also showed similar findings. An RBD binding antibody profile marked by entirely IgM^+^ was found more often in children with MtD (11% vs. 36%, *p* = .02). A year later, the binding antibody profiles changed dramatically. Compared to children with MtD, family members showed higher frequencies for spike and RBD IgG (93% vs. 59%, *p* = .004; 100% vs. 76%, *p* = .006; respectively). Over the year, family members and children with MtD increased the frequency of infections and binding antibody diversity (last two columns, *p* < .05 overall). In summary, these binding antibody profiles indicate that children with MtD were experiencing recent infections at our initial collection, whereas 1 year later, both family members and children with MtD showed higher frequencies of past exposure marked by increasing binding antibody diversity, due to natural infection and vaccination.

### 3.2 Family structures for SARS‐CoV‐2 infections

To understand SARS‐CoV‐2 status and the infection risk posed to the child(ren) with MtD during 2020 and 2021–2022, we represented household structures as spoke diagrams with child(ren) with MtD at the centre, surrounded by other family members (Figure 3). Arrows represent collection at more than one timepoint approximately 1 year apart. Of note, one family (Family 10) had a third collection weeks after their second, due to the appearance of symptoms. Vaccination status and the breakdown of the binding antibody profile were considered while defining seropositive cases. Unvaccinated individuals were considered seropositive if they had binding antibody(ies) against any of the viral proteins tested. Individuals who were found to have nucleocapsid binding antibodies were considered seropositive regardless of vaccination status, as nucleocapsid antibodies cannot arise from vaccination. In 2020–2021, all families (20/20, 100%) had one or more individuals with evidence of infection, and 20/22 (91%) children with MtD showed evidence of infection. In 2021–2022, the numbers remained high with 13/15 (87%) families having at least one individual with evidence of infection; however, children with MtD decreased to 10/17 from the previous year (91% vs. 59%, *p* = .03). This finding was in part due to vaccination. At our initial collection (households *N* = 20, participants *N* = 83), 12/83 (14%) participants had received at least one dose of the COVID‐19 vaccine. At our second collection 1 year later, 41/58 (71%) participants received at least one dose of the COVID‐19 vaccine. Of note, during the first collection period, COVID‐19 vaccines were not yet readily available to the public; therefore, vaccine rates were based on local access rather than compliance. At the second collection, COVID‐19 vaccines were readily available for ages 12 and up and were recently approved for ages 5–12 years (Nov 2021). Therefore, 58 of participants were eligible and 44 had received at least one dose the vaccine, indicating a compliance rate of 76%.

### 3.3 SARS‐CoV‐2 neutralizing antibody titres

Although our initial studies on binding antibodies were important for defining infection status, quantifying neutralizing antibodies are an important step in characterizing the quality of the antibody response and immune protection. Neutralizing antibodies bind to viral particles and interfere with its ability to infect cells. Indeed, SARS‐CoV‐2 neutralizing antibody levels are predictive of immune protection from symptomatic infection.^33^ To help define immune protection, we measured the neutralization of SARS‐CoV‐2 alpha, delta and omicron variants in a pseudovirus neutralization assay (PsVNA) (Figure 3). In 2020, although a large majority of the cohort showed evidence of infection, very few participants had neutralizing antibodies to SARS‐CoV‐2 (Figure 3A). Neutralizing antibodies for the alpha variant, the predominant variant at the time, were present in 15/61 (25%) of family members and 1/22 (4.5%) of children with MtD. By 2021, those numbers had risen for family members to 31/41 (75%, *p* < .00001) and 7/17 (41%, *p* = .01). We next asked how vaccination affected neutralizing antibody levels for the two groups in our cohort (Figure 3B). For family members who were unvaccinated (10/41, 24%), 6/10 (60%) had neutralizing antibodies for the alpha variant, albeit at low levels. Only 1/10 had neutralizing antibody titres for the delta variant, and 2/10 for the omicron variant. Similar to the alpha variant, these levels were very low. For unvaccinated children with MtD (7/17), only 1 individual had neutralizing antibodies to the alpha, delta and omicron variants. Vaccinated family members had neutralizing antibody titres that were 5.5× higher (*p* = .01) for alpha, 2.7× higher for delta (*p* = .04) and 4.3× higher (*p* = .06) for omicron. Vaccinated children with MtD had neutralizing antibody titres that were higher for alpha, delta and omicron; however, these differences were not significant, likely due to small numbers. Also of note, children with MtD tended to have lower levels of neutralizing titres when compared across other family members. However, when compared across paediatric family members, children with MtD had similar neutralizing antibody titres for alpha, delta and omicron variants (Figure 3C). Generally, we found that vaccination results in a more robust and diverse immune response offering protection from SARS‐CoV‐2 infection.

### 3.4 Validation of collection technique for VirScan

Due to the evolving nature of the COVID‐19 pandemic, it was necessary to perform traditional serologic studies of SARS‐CoV‐2. To remove the logistical barriers of performing multiple tests for determining additional viral exposures, we adopted an unbiased multiplex approach. Recently, a comprehensive and validated research platform called VirScan has been developed to query the human viral exposome.^34^ VirScan uses a phage display library containing a synthetic viral proteome to capture reactive antibodies. Next generation sequencing of phages provides a readout of viral peptides which can be assembled into viral proteins, indicating an individual's viral exposome. The whole process can be performed using small blood volumes (μl), making this platform ideal for paediatric research and an important tool in infectious disease research. Although our Neoteryx platform (i.e. capillary blood collection) has been validated for the detection of SARS‐CoV‐2 antibodies,^19^ we wanted to evaluate this collection platform for VirScan analysis. To accomplish this, we collected serum and Neoteryx samples from healthy individuals (*N* = 5) and compared peptide counts and identification of viral infections (Figure S2). Viral peptide counts showed a high degree of correlation between serum and Neoteryx samples (Figure S2A, *r* = .93). Our regression coefficient was also strong, explaining 87% of the variance in the linear model (*r* ^2^ = .87, *p* < .0001). Next, we asked whether the AVARDA would produce similar results for the viral exposome for common respiratory and gastrointestinal viral infections. On average (Figure S2B), there was substantial agreement^35^ between the two sampling methods (89% agreement, *k* = .78) prompting us to use this method for our household serologic study.

### 3.5 Viral infections in households via phage display and immunoprecipitation

During the 2020–2021 period of observation, 15 families (39 family members and 17 children with MtD) were available for VirScan study (Figure 4). Family members experienced 103 viral infections, whereas children with MtD experienced 86 viral infections. Of the 38 different viruses detected, our findings translated to an average of 2.6 infections per family member and 5.1 infections per child with MtD during the study period (approximately 1 year). One participant in Family 10 was found to have antiviral antibodies to the Orf virus, a pox virus associated with sheep or goat exposure (Figure 4). This finding was consistent with the individual's history of international travel.

The top 10 viruses detected by VirScan are listed in Table 2. Enterovirus B (23% family vs. 53% MtD, *p* = .06) and Rhinovirus A (31% family vs. 53% MtD, *p* = .14) were the most common viruses. Interestingly, Enterovirus C was seen in greater frequency in children with MtD (7.7% vs. 47%, *p* = .002), probably due to developing immunity in children. We next examined the most commonly shared viruses between children with MtD and family members. A shared virus was defined as being present in the child with MtD and at least one other family member. Besides being the most frequent virus, Enterovirus B (5/17, 29%) was also the most frequently shared virus, along with Enterovirus C (4/17, 24%). This finding is not surprising as enteroviruses in general are spread by close contact with an infected person via secretions or the faecal–oral route (Table S2). Although SARS‐CoV‐2 was not included in this version of the VirScan library and was covered by our previously discussed serologic testing, SARS‐related coronavirus was detected in five families due to cross‐reactive antibodies. Certainly, select domains of the spike protein are conserved amongst coronaviruses.^36^ Of note, the viral infections documented in the households for this study were similar to a multi‐year cohort of paediatric healthy controls recently published.^37^ Our VirScan and SARS‐CoV‐2 studies indicate that despite adherence to RMBs during the COVID‐19 pandemic by families with a child with MtD,^38^ viral infection is pervasive.

### 3.6 Clinical correlates during the study period

To correlate evidence of frequent viral infections with real‐world patient experience, we next documented caregiver‐reported illness events. To accomplish this, we interviewed caregivers and reviewed medical records (Table 3) to identify timepoints when the child experienced illness and whether a pathogen was identified in a clinical setting. Over the course of the study, 17 children with MtD experienced 34 sick episodes, averaging 2 episodes per child (range 1–5 episodes/child), with 33/34 (97%) presenting with symptoms of an infectious illness. Three children (MtD 4,5,6) did not experience any illnesses during the study. We also noted vaccination status for respiratory illnesses as it may impact severity of illness symptoms, at the second timepoint, 10/17 (59%) had at least one dose of the COVID‐19 vaccination and 13/17 (76%) had received their flu vaccination.

**TABLE 3:** Illness in children with MtD

MtD patient	Phenotype	IPMDS score	COVID‐19 vax	Influenza vax	Antiviral antibodies	Illnesses	Testing	Management	Neurologic changes
1	LS	25	Y	Y	Entero, HHV‐6B, Noro, RSV, Rhino, SARS	Jul 21: F, SZ, PN Sep 21: F, URI Oct 21: F, URI Dec 21: F, PN	StrepA neg, C19 neg, Resp panel neg, CXR pos C19 neg, Resp panel neg, CXR neg C19 neg, Resp panel neg, CXR neg C19 neg, CXR pos	ICU: BiPAP Home Home Hosp	SZ pattern None None SZ pattern
2	LS	21	N	N	Entero, AdV, Noro, SARS	Jun 21: URI, trach inf Jan 22: URI	C19 pos C19 pos	Home: Abx Home	None No change
3	LLS	34	YY	Y	SARS	Oct 21: URI Jan 22: PN	None C19 pos, Resp panel neg, CXR pos	Home: Abx ICU: remdesivir, IVIG, steroids	Gait and fine motor Dystonia
4	LS	17	N	N	AdV, SARS	None	NA	NA	NA
5	LS	17	YYY	Y	Entero, Noro, SARS	None	NA	NA	NA
6	MtD NOS	12	N	N	Entero, Rhino, SARS	None	NA	NA	NA
7	LS	48	N	N	Entero, HHV‐6B, RSV, SARS	Oct 21: URI, Resp	C19 neg, RSV pos, CXR neg	ICU: trach, vent, IVIG	New SZ activity
9	LS	6	N	Y	Entero, AdV, Rhino, SARS	Jun 21: F, NV Oct 21: URI Nov 21: URI, PN	C19 neg C19 neg Resp panel?	Home Home Home	None None None
10	LS	15	Y	Y	SARS	Nov 21: URI Dec 21: F, URI	StrepA neg, C19 neg, Flu neg C19 pos	Home Home	None None
11	MERRF	21	YYY	Y	Entero, SARS	Oct 21: ST	StrepA pos, C19 neg, Resp panel neg	Home: Abx	None
12	MtD NOS	11	N	Y	Entero, Flu, Rhino, SARS	Jul 21: F, ST, SN Sep 21: URI, SN Oct 21: URI, SN Dec 21: URI, SN Jan 22: F, URI, ST	Resp panel? C19 neg, Resp panel neg C19 neg, Resp panel neg C19 neg, Resp panel neg C19 pos	Home Home Home Home Home	Ataxia, speech changes Nystagmus Nystagmus Nystagmus None
13	MELAS	2	YYY	Y	HMPV, Flu, Rhino, SARS	Jan 22: F, URI	None	Home	None
14	MELAS	8	YYY	Y	Entero, Astro, HMPV, RSV, Rhino	Jan 22: F, URI, V	C19 neg	Home	None
16	MtD NOS	25	YY	Y	Entero, Flu, Rhino, SARS	Jul 21: URI Oct 21: URI	None C19 neg	Home Home	None None
17	MtD NOS	15	YY	Y	Entero, AdV, Flu, Rhino, SARS	Jul 21: URI Oct 21: ST, URI	None None	Home Home	None None
19	LS	13	N	Y	AdV, RSV, Rhino, SARS	Apr 21: F, URI May 21: URI, AOM Sep 21: F, URI, AOM, PNA Dec 21: URI, AOM Jan 22: URI, AOM	C19 neg None Resp panel neg C19 neg, Resp panel neg C19 neg, Resp panel neg	Home: Abx Home: Abx Home: Abx Home: Abx Home: Abx	None None None None None
21	LS	47	Y	Y	Entero, Rhino, SARS	6/21: UTI 7/21: F, URI 7/21: F, URI 1/22: Neuro storm	C19 neg, Resp panel neg, Bld cx neg C19 neg, Resp panel neg C19 neg, Resp panel neg C19 neg, Resp panel neg	Hosp: Abx Home Home Hosp	None None None Facial weakness, rigidity

*Note*: Children with MtD were followed for approximately 1 year during the winter seasons of the COVID‐19 pandemic. Illnesses episodes were recorded via caregiver interviews and a review of patient records. Changes in neurologic status were recorded for illness episodes.

Abbreviations: Abx, antibiotics; AOM, acute otitis media; BiPAP, bi‐level positive airway pressure; F, fever; HHV‐6B, human herpes virus 6B; Hosp, hospital; ICU, intensive care unit; IVIG, intravenous immunoglobulins; LLS, Leigh‐like syndrome; LS, Leigh syndrome; MELAS, mitochondrial encephalopathy lactic acidosis and stroke; MERRF, myoclonus epilepsy with ragged‐red fibres; MtD NOS, mitochondrial disease not otherwise specified; N, no vaccinations; NA, not applicable; Neuro storm, neurologic storm; NV, nausea/vomiting; PN, pneumonia; Resp, respiratory distress; RSV, respiratory syncytial virus; SN, sinusitis; ST, sore throat; SZ, seizures; trach inf, tracheostomy infection; URI, upper respiratory illness; vax, vaccination; vent, ventilator; Y, single vaccination; YY, two vaccinations; YYY, two vaccinations.

To understand the relationship between viral infections and illness episodes, we calculated the crude attack rate using the number of illness events divided by the number of viral infections detected by serologic testing. The crude attack rate for illness events in children with MtD was 35%. Although this measure was imperfect, we interpreted these data as indicating that a proportion of viral infections were subclinical or asymptomatic.

For each illness event experience by the child with MtD, we also assessed the treatment disposition for the illness episode. We found 6/34 (18%) episodes required hospitalization, 4/6 (67%) of which were ICU admissions. For the hospitalization episodes, children with MtD displayed higher IPMDS‐D1 scores (median 14 vs. 40.5, *p* < .003, Figure S3). Not surprisingly, the most common symptoms recorded included upper respiratory tract illness (URI, 26/64 symptoms, 40%) and fever (F, 13/64 symptoms, 20%). Viral and or bacterial testing was performed in 28/34 episodes (82%) and identified an organism in only 7 cases (7/28, 25%). Identified organisms included Streptococcus A (1/34, 2.9%), respiratory syncytial virus (1/34, 2.9%) and COVID‐19 (5/34, 15%). All children with MtD showed serologic evidence of viral infections via our studies, some of which were not covered by clinical testing (e.g. human herpes virus 6B [HHV‐6B]).

**FIGURE 2:**
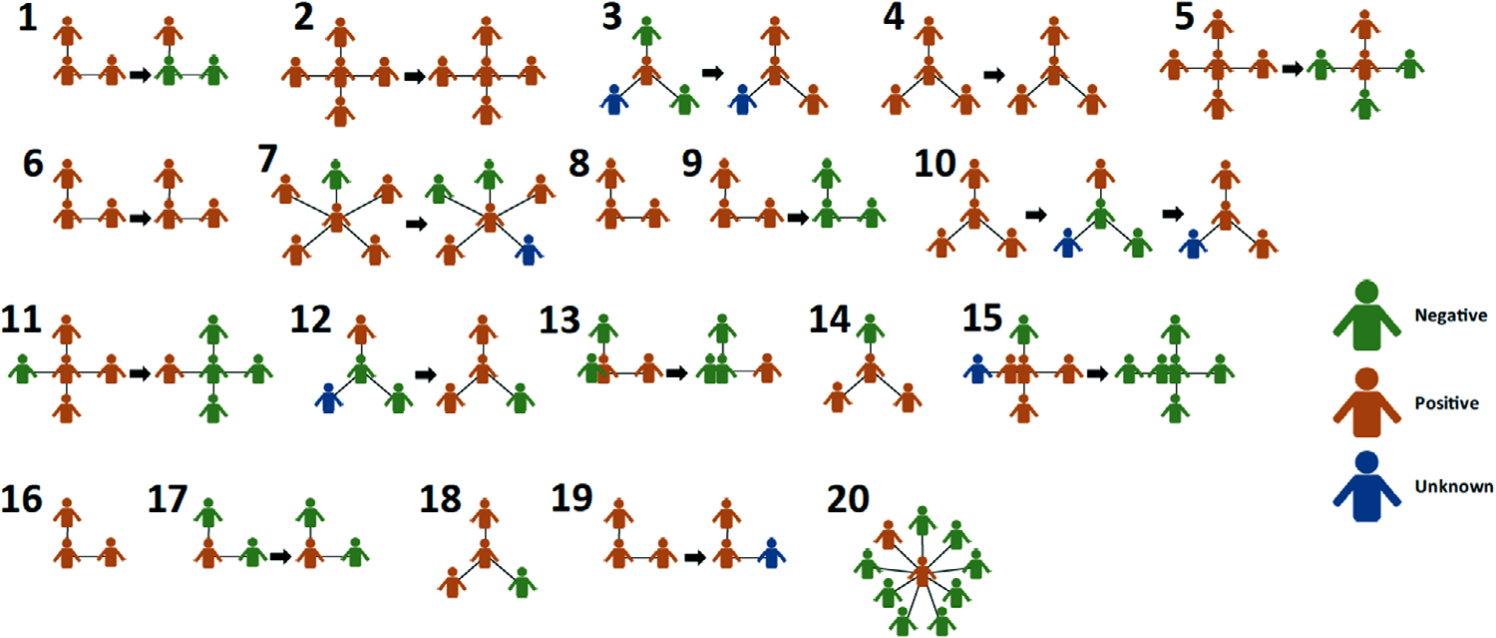
2020–2021 spoke diagrams and SARS‐CoV‐2 serology. To highlight the relationship between individuals in the household (*N* = 20) and the child(ren) with mitochondrial disease (*N* = 17) which are represented as a spoke diagram. The child(ren) with mitochondrial disease (MtD) is located at the centre and is connected to household members by spokes. Viral nucleocapsid, spike protein and receptor binding domain (receptor‐binding domain [RBD]) serologies were performed as outlined in the methods. Seropositive status was defined by the binding antibody profiles, taking into account vaccination status. Individuals who had nucleocapsid IgG were considered positive regardless of vaccination status. Households that had two collections (2020 and 2021) are connected by an arrow. Households are also numbered 1–20, and SARS‐CoV‐2 serology status is indicated by the colour key.

Interestingly, during our initial sample collection, 20/22 children with MtD showed evidence of COVID‐19 exposure and were undiagnosed and largely asymptomatic (Figure 2). By our second collection, five symptomatic cases occurred (Table 2). All children who had positive clinical testing for COVID‐19 were also identified by our home‐based testing, lending validity to our methods. One COVID‐19 positive patient (MtD 3) developed severe pneumonia and was admitted to the ICU for care, where they received intravenous immune globulin, remdesivir and steroids.

**FIGURE 3:**
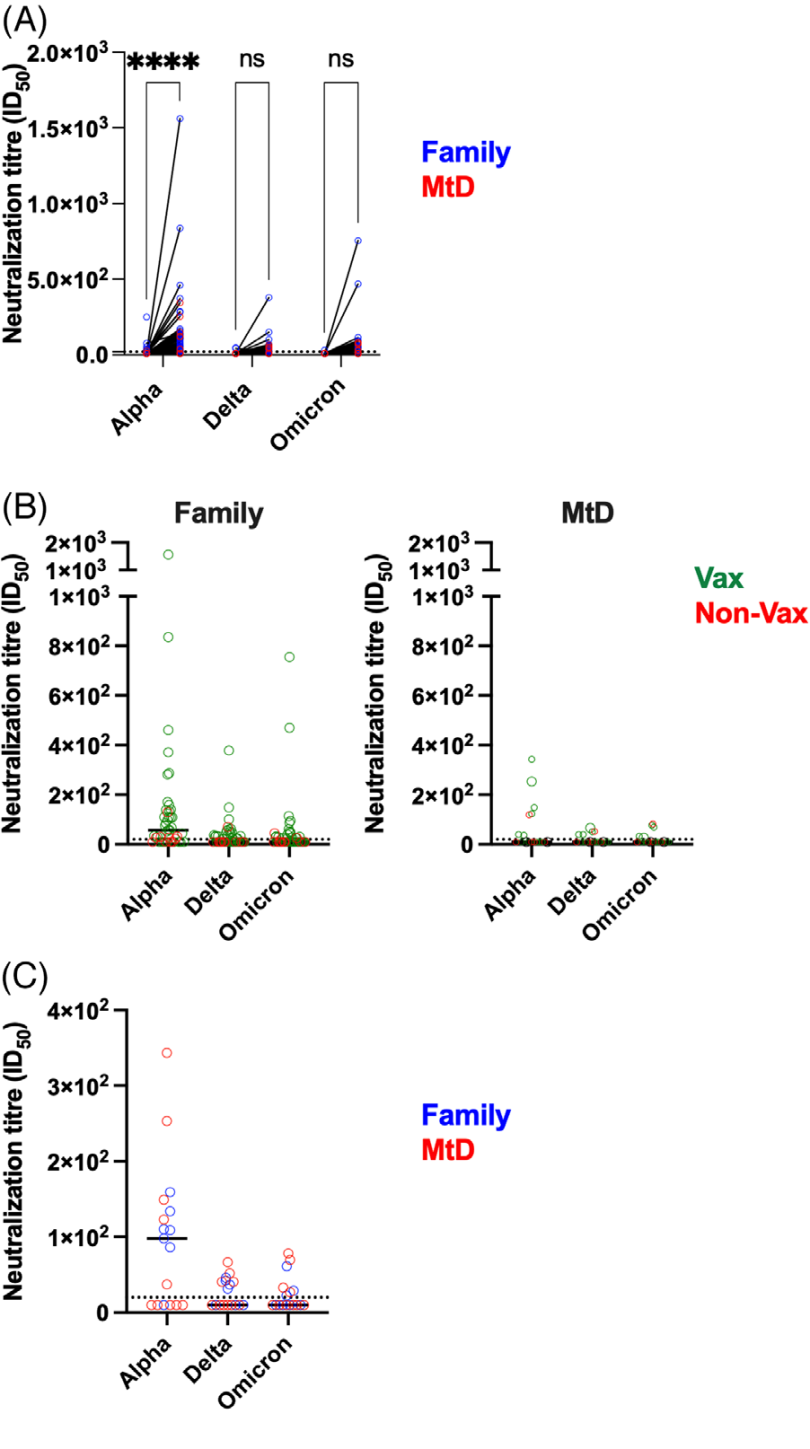
SARS‐CoV‐2 neutralizing antibodies. SARS‐CoV‐2 PsVNA was performed on capillary blood samples eluted from the microsampling collection apparatus as shown before. Neutralizing antibodies are a functional readout of the ability to block viral biologic activity. Neutralizing antibodies against the pseudovirions expressing spike proteins from alpha, delta and omicron variants were determined. Assays were performed in duplicate, and the 50% neutralization titre was calculated. Controls included cells only, virus without any antibody and positive sera. (A) Change (2020–2021) in neutralizing antibody titres for family members (blue) and children with mitochondrial disease (MtD) (red) between first (2020) and second (2021) collections. (B) Neutralizing antibody titres for vaccinated (green) and unvaccinated (red) participants. (C) Neutralization titres for children with MtD (red) and paediatric family members (blue). Two‐way ANOVA with mixed‐effects analysis. ****p < .0001

**FIGURE 4:**
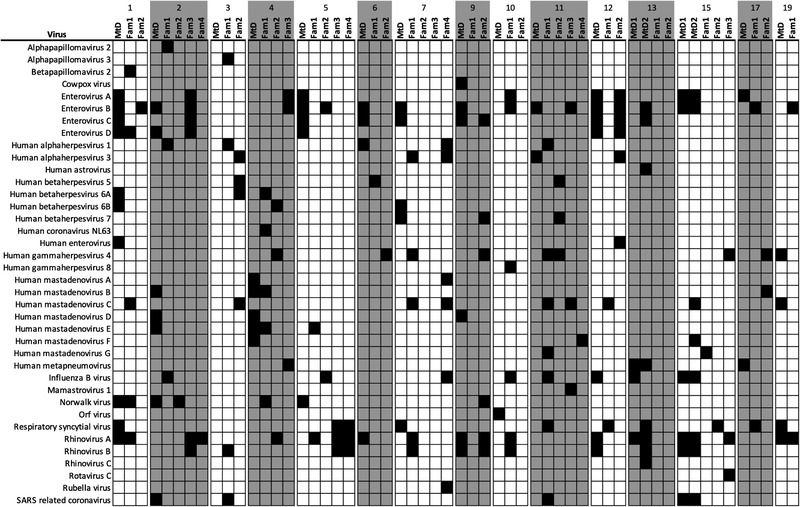
Family heat maps for viral infections. Capillary blood samples were collected and eluted as above. Eluate was used to probe for antiviral antibodies using phage display and immunoprecipitation sequencing (VirScan). Viral infections were called via a deconvolution algorithm (AntiViral Antibody Response Deconvolution Algorithm [AVARDA]). Dark box indicates exposure to the indicated virus (FDR < .05). Virus exposures for the period of study were determined by comparing antiviral antibody profiles between the first and second collections. Families are numbered according to Figure 2. Fam, family members; MtD, mitochondrial disease

A previous study found that children with MtD may experience sequelae, including neurologic events following infection.^12^ Neurological events were captured by caregiver interview. In our cohort, 5/17 (29%) children with MtD (mean age = 4.4 years, range = 3–5 years) experienced neurologic events with their illness. Three children (MtD 1, 3, 12) experienced two or more separate neurologic events, bringing the total to 10/34 (29%) events overall. All these children continue to be evaluated beyond the study period for the persistence of these changes. An infectious agent was clinically defined for two of the five children with neurologic events and included SARS‐CoV‐2 (MtD 3) and respiratory syncytial virus (RSV) (MtD 7). As stated before, antiviral antibody profiles revealed viral infections for all children with MtD and even included neurotropic viruses (e.g. HHV‐6B, HHV‐7) known to cause or exacerbate neurologic conditions.^39^ Overall, our data suggests that deep phenotyping of the viral exposome using VirScan following an infectious illnesses may be useful in identifying potential pathogens not detected on clinical testing.^37^

## 4 DISCUSSION

### 4.1 Summary

From a clinical perspective, managing children with MtD can be challenging, due to the risk of disease progression and cumulative disability following viral infection.^12^, ^40^, ^41^ The mechanisms by which children with MtD deteriorate during infection are still being elucidated and include abnormal cell stress responses^42^ and cytokine‐mediated toxicity,^43^ suggesting that disease progression following viral infection represents a failure of allostasis.^44^ Therefore, understanding the number and breadth of viral infections (i.e. viral exposome) in children with MtD is an important first step in defining the mechanisms behind these deleterious host–pathogen interactions. As person‐to‐person transmission of viral infections can occur in the household, we sought to define the viral exposome in households with an at‐risk child with MtD. Besides having MtD, we focused on children with additional comorbidities associated with adverse outcomes during or following viral infection. To accomplish this, we conducted a home‐based serologic study of antiviral antibodies for COVID‐19 and other viruses during the pandemic. Overall, we found that children with MtD experienced numerous common viral infections including SARS‐CoV‐2, several of which resulted in illness, hospitalization and exacerbation of neurologic symptoms. Fortunately, there were no deaths in our cohort.

### 4.2 SARS‐CoV‐2

The COVID‐19 pandemic continues to present challenges to medically vulnerable paediatric communities, including children with MtD. During our initial collection in 2020, despite high penetration into families, most infections were undiagnosed and either oligo‐ or asymptomatic. A careful assessment of antibody profiles in children with MtD revealed a history of more recent infections, due to the predominance of the IgM isotype for anti‐SARS‐CoV‐2 antibodies. Furthermore, neutralizing antibody titres were essentially non‐existent for all participants. By our second collection in 2021, despite the persistence of high numbers of exposed individuals in households, the serologic and clinical profiles were markedly different. First, the absorbances for SARS‐CoV‐2 antibodies (IgG and IgM) appreciably increased for family members and children with MtD indicating a more robust immune response. Second, although the number of vaccinated individuals increased during this time, natural infection was also a contributing factor; the number of IgG^+^ nucleocapsid antibody cases also had risen similarly for both family members and children with MtD. Third, the number of individuals who were positive for neutralizing antibodies also surged in 2021, indicating a more robust immune response compared to the previous year.

Besides the effects of vaccination, the differences between these two timepoints may also be related to household behaviours. Recently, we showed that families affected by MtD display high levels of adherence to RMBs during the pandemic, likely due to fear of potential sequelae associated with infection.^38^ Although our data is consistent with households being highly adherent in 2020, as the COVID‐19 pandemic persisted, there was the possibility of ‘pandemic fatigue’ setting in by 2021. This may manifest as (1) demotivation in continuing to adhere to RMB's and (2) boredom regarding pandemic‐related information.^45^ Indeed, although mask wearing has seen a general increase over time, high‐cost and sensitizing behaviours involving physical distancing or avoidance of contact declined over time.^46^, ^47^ In our cohort, we hypothesize that early implementation of RMBs in 2020 led to attenuated SARS‐CoV‐2 infections that were enough to produce serologic markers of exposure but lacked sufficient potency to cause illness or robust immune responses.^48^ By 2021, due to a decline in RMBs adherence and persistence of the pandemic, more substantial exposures occurred resulting in more episodes of clinical illness and robust markers of exposure. The marked changes in antibody profiles are likely due to a combination of vaccination and natural infection. Certainly, a combination of infection acquired immunity and vaccination can convey more robust immune responses and long‐term protection.^49^

As vaccines are now available for children, an added concern has emerged. Although vaccination against SARS‐CoV‐2 can prevent infection, ameliorate COVID‐19 severity^50^ and reduce household transmission,^51^ we have reported significant vaccine hesitancy for children with MtD due to the perceived risk of disease progression.^52^ Although we strongly advocate for vaccination in this high‐risk population, the high degree of household seropositivity seen in our spoke diagrams reinforces the need for concomitantly pursuing household members for ring vaccination. Ring vaccination aims to vaccinate household and other frequent contacts of a vulnerable individual, thereby reducing the risk of exposure to a specific infection. In fact, ring vaccination has been considered as a strategy for the evaluation of vaccine efficacy and effectiveness.^53^ For the MtD community, ring vaccination may be an important strategy to bridge to vaccination. Future studies will not only need to address the uptake and efficacy/effectiveness of vaccination for patients with MtD, but also the value of ring vaccination in the MtD community.

### 4.3 Other viral infections

The COVID‐19 pandemic and consequent implementation of RMBs initially reduced the transmission of viral respiratory pathogens; however, many have seen a return or modulation of their seasonality (Table S2).^13^, ^14^ Between March and April 2020, children experienced a rapid decline in acute respiratory illnesses across seven US cities, particularly for RSV and influenza. However, beginning in June 2020, rhinoviruses and enteroviruses began to return. In the following year, although influenza and human metapneumovirus circulated at historic lows through May 2021, April was marked by a rise in RSV activity. Human coronaviruses, parainfluenza viruses and respiratory adenoviruses also saw upsurges beginning in January/February 2021. Besides SARS‐CoV‐2, our data indicates that children with MtD experienced up to five viral infections over the 2020–2021 season, including enterovirus, rhinovirus, RSV and influenza. For Enterovirus B, antibody profiles were shared with another family member 29% of the time, indicating that although the household remains an important risk factor, other circumstances influenced viral infections. One such factor is ‘pandemic fatigue’ mentioned before. Another factor may be the shift in learning during the pandemic; the 2021 return to school saw widespread variation in RMBs which impacted infection rates.^54^

### 4.4 Illnesses

The breadths of viral infections in children with MtD were similar to a previously reported cohort of healthy children.^37^ Therefore, although children with MtD have similar viral infections, they are at greater risk of adverse clinical outcomes from these host–pathogen interactions. Though having MtD is clearly a risk factor, a major question remains regarding whether specific viruses are associated with disease progression. Even though children with MtD experienced a considerable number of viral infections, about 1/3 resulted in clinical illness. This previously unreported finding in children with MtD confirms that not all viral infections lead to significant illness or disease progression. Future studies focusing on more frequent sampling following viral illnesses will help us identify viruses that may be associated with progression to clinical illness and decline.

As mentioned previously, 2020 was marked by asymptomatic infection for SARS‐CoV‐2 in our cohort. In 2021, four children with MtD developed COVID‐19, with one child developing COVID‐19 on two separate occasions. Although most were treated at home, one was admitted to the ICU and received remdesivir, intravenous immune globulin and steroids for COVID‐19. This child experienced dystonia following his infection. Indeed, neurologic sequelae following viral infection are not uncommon in MtD.^12^ In our cohort, ≈30% of children with MtD developed neurologic complications following viral infection, and COVID‐19 was not associated with more frequent neurological complications than other viruses. Another patient experienced a neurologic deterioration with RSV, leading to the development of new seizure activity and respiratory failure requiring a tracheostomy. This child has Leigh syndrome due to a deleterious variant in *ATP6*, an MtD marked by viral infection‐induced deterioration in respiratory function due to brainstem damage. An outcome like this is not unanticipated, and often feared, for this type of MtD.

One final consideration regarding illnesses and viral infections in children with MtD includes potential long‐lasting effects that may contribute to disease progression. Although unknown in MtD, evidence suggests that SARS‐CoV‐2 can impair mitochondria potentially leading to disease exacerbation.^55^ Individuals with COVID‐19 can also exhibit neurologic signs, indicating CNS infection^56^, ^57^, ^58^; a finding supported by human iPSC cerebral organoid models and autopsy studies.^56^, ^59^ Furthermore, CNS infection can persist, lasting up to 230 days.^56^ Overall, the potential for neuroinvasion of SARS‐CoV‐2 raises the concern for acute or chronic neurodegeneration in children with MtD via damaging neuroinflammation.^60^ Similar concerns are shared for HHV‐6B.^61^ HHV‐6B infects the CNS and enters a state of latency. This herpesvirus can also integrate into the host genome (i.e. chromosomally integrated HHV‐6B, ciHHV‐6B) at the telomeres allowing it to persist in the human host indefinitely and even pass through the germline. Under immune surveillance, these infections are asymptomatic. However, HHV‐6 has been associated with CNS pathology in the context of stressors and genetic risk factors.^61^ In addition, this virus has been implicated in multiple neurological conditions, including encephalitis, epilepsy, febrile seizures, as well as neurodegenerative disorders like multiple sclerosis and Alzheimer's disease where neuroinflammation plays a role.^39^ In summary, in children with MtD, certain viruses may have immediate and long‐lasting effects on disease pathophysiology.

## 5 CONCLUSIONS

In conclusion, our study highlights the penetration of viral infections into a neurologically vulnerable community despite adherence to RMBs. More importantly, our understanding of the natural history of MtD, particularly the relationship with viral infection, has advanced. Future studies will help elucidate which viruses or variants cause neurologic decline and which do not, with the goal of improving clinical care. Generally speaking, our at‐home sampling platform and study objectives also serve as a template for overcoming research barriers and accessing understudied populations during and beyond the pandemic.

## DISCLAIMER

The NIH, its officers and employees do not recommend or endorse any company, product or service.

## CONFLICTS OF INTEREST

The authors have no conflicts of interest to declare.

## Supporting information

Supporting Information

## References

[1] Carabin H , Gyorkos TW , Soto JC , Penrod J , Joseph L , Collet JP . Estimation of direct and indirect costs because of common infections in toddlers attending day care centers. Pediatrics. 1999;103(3):556‐564. doi: 10.1542/peds.103.3.556 10049956

[2] Gonzales R , Malone DC , Maselli JH , Sande MA . Excessive antibiotic use for acute respiratory infections in the United States. Clin Infect Dis. 2001;33(6):757‐762. doi: 10.1086/322627 11512079

[3] Kvaerner KJ , Nafstad P , Jaakkola JJ . Upper respiratory morbidity in preschool children: a cross‐sectional study. Arch Otolaryngol Head Neck Surg. 2000;126(10):1201‐1206. doi: 10.1001/archotol.126.10.1201 11031406

[4] Graham NM . The epidemiology of acute respiratory infections in children and adults: a global perspective. Epidemiol Rev. 1990;12:149‐178. doi: 10.1093/oxfordjournals.epirev.a036050 2286216

[5] Rosenfeldt V , Vesikari T , Pang XL , Zeng SQ , Tvede M , Paerregaard A . Viral etiology and incidence of acute gastroenteritis in young children attending day‐care centers. Pediatr Infect Dis J. 2005;24(11):962‐965. doi: 10.1097/01.inf.0000183748.41027.a4 16282929

[6] Wilhelmi I , Roman E , Sanchez‐Fauquier A . Viruses causing gastroenteritis. Clin Microbiol Infect. 2003;9(4):247‐262. doi: 10.1046/j.1469-0691.2003.00560.x 12667234 PMC7129320

[7] Centers for Disease C, Prevention . Influenza vaccination practices of physicians and caregivers of children with neurologic and neurodevelopmental conditions – United States, 2011–12 influenza season. MMWR Morb Mortal Wkly Rep. 2013;62(36):744‐746. 24025756 PMC4585574

[8] Ng YS , Turnbull DM . Mitochondrial disease: genetics and management. J Neurol. 2016;263(1):179‐191. doi: 10.1007/s00415-015-7884-3 26315846 PMC4723631

[9] Naviaux RK , Nyhan WL , Barshop BA , et al. Mitochondrial DNA polymerase gamma deficiency and mtDNA depletion in a child with Alpers' syndrome. Ann Neurol. 1999;45(1):54‐58. 9894877 10.1002/1531-8249(199901)45:1<54::aid-art10>3.0.co;2-b

[10] Tarasenko TN , Pacheco SE , Koenig MK , et al. Cytochrome c oxidase activity is a metabolic checkpoint that regulates cell fate decisions during T cell activation and differentiation. Cell Metab. 2017;25(6):1254‐1268.e7. doi: 10.1016/j.cmet.2017.05.007 28591633 PMC5562283

[11] Eom S , Lee HN , Lee S , et al. Cause of death in children with mitochondrial diseases. Pediatr Neurol. 2017;66:82‐88. doi: 10.1016/j.pediatrneurol.2016.10.006 27843091

[12] Edmonds JL , Kirse DJ , Kearns D , Deutsch R , Spruijt L , Naviaux RK . The otolaryngological manifestations of mitochondrial disease and the risk of neurodegeneration with infection. Arch Otolaryngol Head Neck Surg. 2002;128(4):355‐362. doi: 10.1001/archotol.128.4.355 11926907

[13] Haddadin Z , Schuster JE , Spieker AJ , et al. Acute respiratory illnesses in children in the SARS‐CoV‐2 pandemic: prospective multicenter study. Pediatrics. 2021;148(2):e2021051462. doi: 10.1542/peds.2021-051462 33986150 PMC8338906

[14] Olsen SJ , Winn AK , Budd AP , et al. Changes in influenza and other respiratory virus activity during the COVID‐19 pandemic – United States. MMWR Morb Mortal Wkly Rep. 2021;70(29):1013‐1019. doi: 10.15585/mmwr.mm7029a1 34292924 PMC8297694

[15] Madewell ZJ , Yang Y , Longini IM Jr , Halloran ME , Dean NE . Factors associated with household transmission of SARS‐CoV‐2: an updated systematic review and meta‐analysis. JAMA Netw Open. 2021;4(8):e2122240. doi: 10.1001/jamanetworkopen.2021.22240 34448865 PMC8397928

[16] Madewell ZJ , Yang Y , Longini IM Jr , Halloran ME , Dean NE . Household secondary attack rates of SARS‐CoV‐2 by variant and vaccination status: an updated systematic review and meta‐analysis. JAMA Netw Open. 2022;5(4):e229317. doi: 10.1001/jamanetworkopen.2022.9317 35482308 PMC9051991

[17] Savage R , Whelan M , Johnson I , et al. Assessing secondary attack rates among household contacts at the beginning of the influenza A (H1N1) pandemic in Ontario, Canada, April‐June 2009: a prospective, observational study. BMC Public Health. 2011;11:234. doi: 10.1186/1471-2458-11-234 21492445 PMC3095560

[18] Koene S , Hendriks JCM , Dirks I , et al. International paediatric mitochondrial disease scale. J Inherit Metab Dis. 2016;39(5):705‐712. doi: 10.1007/s10545-016-9948-7 27277220 PMC4987390

[19] Kalish H , Klumpp‐Thomas C , Hunsberger S , et al. Undiagnosed SARS‐CoV‐2 seropositivity during the first 6 months of the COVID‐19 pandemic in the United States. Sci Transl Med. 2021;13(601):eabh3826. doi: 10.1126/scitranslmed.abh3826 34158410 PMC8432952

[20] Esposito D , Mehalko J , Drew M , et al. Optimizing high‐yield production of SARS‐CoV‐2 soluble spike trimers for serology assays. Protein Expr Purif. 2020;174:105686. doi: 10.1016/j.pep.2020.105686 32504802 PMC7271859

[21] Mehalko J , Drew M , Snead K , et al. Improved production of SARS‐CoV‐2 spike receptor‐binding domain (RBD) for serology assays. Protein Expr Purif. 2021;179:105802. doi: 10.1016/j.pep.2020.105802 33248226 PMC7687410

[22] Tang J , Ravichandran S , Lee Y , et al. Antibody affinity maturation and plasma IgA associate with clinical outcome in hospitalized COVID‐19 patients. Nat Commun. 2021;12(1):1221. doi: 10.1038/s41467-021-21463-2 33619281 PMC7900119

[23] Ravichandran S , Coyle EM , Klenow L , et al. Antibody signature induced by SARS‐CoV‐2 spike protein immunogens in rabbits. Sci Transl Med. 2020;12(550):eabc3539. doi: 10.1126/scitranslmed.abc3539 32513867 PMC7286538

[24] Neerukonda SN , Vassell R , Herrup R , et al. Establishment of a well‐characterized SARS‐CoV‐2 lentiviral pseudovirus neutralization assay using 293T cells with stable expression of ACE2 and TMPRSS2. PLoS One. 2021;16(3):e0248348. doi: 10.1371/journal.pone.0248348 33690649 PMC7946320

[25] Tang J , Novak T , Hecker J , et al. Cross‐reactive immunity against the SARS‐CoV‐2 Omicron variant is low in pediatric patients with prior COVID‐19 or MIS‐C. Nat Commun. 2022;13(1):2979. doi: 10.1038/s41467-022-30649-1 35624101 PMC9142524

[26] Bellusci L , Grubbs G , Srivastava P , et al. Neutralization of SARS‐CoV‐2 Omicron after vaccination of patients with myelodysplastic syndromes or acute myeloid leukemia. Blood. 2022;139(18):2842‐2846. doi: 10.1182/blood.2022016087 35344579 PMC8964006

[27] Ravichandran S , Tang J , Grubbs G , et al. SARS‐CoV‐2 immune repertoire in MIS‐C and pediatric COVID‐19. Nat Immunol. 2021;22(11):1452‐1464. doi: 10.1038/s41590-021-01051-8 34611361

[28] Mohan D , Wansley DL , Sie BM , et al. PhIP‐Seq characterization of serum antibodies using oligonucleotide‐encoded peptidomes. Nat Protoc. 2018;13(9):1958‐1978. doi: 10.1038/s41596-018-0025-6 30190553 PMC6568263

[29] Monaco DR , Kottapalli SV , Breitwieser FP , et al. Deconvoluting virome‐wide antiviral antibody profiling data. eBiomedicine. 2022;75:103747. 34922324 10.1016/j.ebiom.2021.103747 PMC8688874

[30] Team CC‐R . Severe outcomes among patients with coronavirus disease 2019 (COVID‐19) – United States, February 12‐March 16, 2020. MMWR Morb Mortal Wkly Rep. 2020;69(12):343‐346. doi: 10.15585/mmwr.mm6912e2 32214079 PMC7725513

[31] McMichael TM , Clark S , Pogosjans S , et al. COVID‐19 in a long‐term care facility – King County, Washington, February 27‐March 9, 2020. MMWR Morb Mortal Wkly Rep. 2020;69(12):339‐342. doi: 10.15585/mmwr.mm6912e1 32214083 PMC7725515

[32] Vezzani A , Fujinami RS , White HS , et al. Infections, inflammation and epilepsy. Acta Neuropathol. 2016;131(2):211‐234. doi: 10.1007/s00401-015-1481-5 26423537 PMC4867498

[33] Khoury DS , Cromer D , Reynaldi A , et al. Neutralizing antibody levels are highly predictive of immune protection from symptomatic SARS‐CoV‐2 infection. Nat Med. 2021;27(7):1205‐1211. doi: 10.1038/s41591-021-01377-8 34002089

[34] Xu GJ , Kula T , Xu QK , et al. Comprehensive serological profiling of human populations using a synthetic human virome. Science. 2015;348(6239):aaa0698. doi: 10.1126/science.aaa0698 26045439 PMC4844011

[35] McHugh ML . Interrater reliability: the kappa statistic. Biochem Med (Zagreb). 2012;22(3):276‐282. 23092060 PMC3900052

[36] Lei KC , Zhang XD . Conservation analysis of SARS‐CoV‐2 spike suggests complicated viral adaptation history from bat to human. Evol Med Public Health. 2020;2020(1):290‐303. doi: 10.1093/emph/eoaa041 33372198 PMC7665476

[37] Monaco DR , Kottapalli SV , Breitwieser FP , et al. Deconvoluting virome‐wide antibody epitope reactivity profiles. eBioMedicine. 2022;75:103747. doi: 10.1016/j.ebiom.2021.103747 34922324 PMC8688874

[38] Gordon‐Lipkin E , Kruk S , Thompson E , et al. Risk mitigation behaviors to prevent infection in the mitochondrial disease community during the COVID19 pandemic. Mol Genet Metab Rep. 2021;30:100837. doi: 10.1016/j.ymgmr.2021.100837 34956836 PMC8683364

[39] Santpere G , Telford M , Andres‐Benito P , Navarro A , Ferrer I . The presence of human herpesvirus 6 in the brain in health and disease. Biomolecules. 2020;10(11):1520. doi: 10.3390/biom10111520 33172107 PMC7694807

[40] Lee WS , Sokol RJ . Mitochondrial hepatopathies: advances in genetics, therapeutic approaches, and outcomes. J Pediatr. 2013;163(4):942‐948. doi: 10.1016/j.jpeds.2013.05.036 23810725 PMC3934633

[41] Parikh S , Saneto R , Falk MJ , et al. A modern approach to the treatment of mitochondrial disease. Curr Treat Options Neurol. 2009;11(6):414‐430. doi: 10.1007/s11940-009-0046-0 19891905 PMC3561461

[42] Suomalainen A , Battersby BJ . Mitochondrial diseases: the contribution of organelle stress responses to pathology. Nat Rev Mol Cell Biol. 2018;19(2):77‐92. doi: 10.1038/nrm.2017.66 28792006

[43] Jestin M , Kapnick SM , Tarasenko TN , et al. Mitochondrial disease disrupts hepatic allostasis and lowers the threshold for immune‐mediated liver toxicity. Mol Metab. 2020;37:100981. doi: 10.1016/j.molmet.2020.100981 32283081 PMC7167504

[44] Tarasenko TN , McGuire PJ . The liver is a metabolic and immunologic organ: a reconsideration of metabolic decompensation due to infection in inborn errors of metabolism (IEM). Mol Genet Metab. 2017;121(4):283‐288. doi: 10.1016/j.ymgme.2017.06.010 28666653 PMC5553615

[45] Cuadrado E , Maldonado MA , Tabernero C , Arenas A , Castillo‐Mayen R , Luque B . Construction and validation of a brief pandemic fatigue scale in the context of the coronavirus‐19 Public Health crisis. Int J Public Health. 2021;66:1604260. doi: 10.3389/ijph.2021.1604260 34566554 PMC8461461

[46] Petherick A , Goldszmidt R , Andrade EB , et al. A worldwide assessment of changes in adherence to COVID‐19 protective behaviours and hypothesized pandemic fatigue. Nat Hum Behav. 2021;5(9):1145‐1160. doi: 10.1038/s41562-021-01181-x 34345009

[47] Crane MA , Shermock KM , Omer SB , Romley JA . Change in reported adherence to nonpharmaceutical interventions during the COVID‐19 pandemic, April‐November 2020. JAMA. 2021;325(9):883‐885. doi: 10.1001/jama.2021.0286 33480971 PMC7823422

[48] Hausdorff WP , Flores J . Low‐dose and oral exposure to SARS‐CoV‐2 may help us understand and prevent severe COVID‐19. Int J Infect Dis. 2021;103:37‐41. doi: 10.1016/j.ijid.2020.11.171 33227512 PMC7678432

[49] Hall V , Foulkes S , Insalata F , et al. Protection against SARS‐CoV‐2 after Covid‐19 vaccination and previous infection. N Engl J Med. 2022;386(13):1207‐1220. doi: 10.1056/NEJMoa2118691 35172051 PMC8908850

[50] Polack FP , Thomas SJ , Kitchin N , et al. Safety and efficacy of the BNT162b2 mRNA Covid‐19 vaccine. N Engl J Med. 2020;383(27):2603‐2615. doi: 10.1056/NEJMoa2034577 33301246 PMC7745181

[51] Singanayagam A , Hakki S , Dunning J , et al. Community transmission and viral load kinetics of the SARS‐CoV‐2 delta (B.1.617.2) variant in vaccinated and unvaccinated individuals in the UK: a prospective, longitudinal, cohort study. Lancet Infect Dis. 2021;22:183‐195. doi: 10.1016/S1473-3099(21)00648-4 34756186 PMC8554486

[52] Gordon‐Lipkin E , Kruk S , Thompson E , et al. Vaccine hesitancy toward the COVID19 vaccine and attitudes toward ring vaccination by caregivers of children with mitochondrial disease. Ann Neurol. 2021;90:S111‐S111.

[53] Kucharski AJ , Eggo RM , Watson CH , Camacho A , Funk S , Edmunds WJ . Effectiveness of ring vaccination as control strategy for Ebola virus disease. Emerg Infect Dis. 2016;22(1):105‐108. doi: 10.3201/eid2201.151410 26691346 PMC4696719

[54] Chernozhukov V , Kasahara H , Schrimpf P . The association of opening K‐12 schools with the spread of COVID‐19 in the United States: county‐level panel data analysis. Proc Natl Acad Sci USA. 2021;118(42):e2103420118. doi: 10.1073/pnas.2103420118 34642247 PMC8545468

[55] Ganji R , Reddy PH . Impact of COVID‐19 on mitochondrial‐based immunity in aging and age‐related diseases. Front Aging Neurosci. 2020;12:614650. doi: 10.3389/fnagi.2020.614650 33510633 PMC7835331

[56] Chertow D , Stein S , Ramelli S , et al. SARS‐CoV‐2 infection and persistence throughout the human body and brain. 20 December 2021, PREPRINT (Version 1) available at Research Square. doi: 10.21203/rs.3.rs-1139035/v1

[57] Yates D . A CNS gateway for SARS‐CoV‐2. Nat Rev Neurosci. 2021;22(2):74‐75. doi: 10.1038/s41583-020-00427-3 PMC7781172 33398138

[58] Yong SJ . Persistent brainstem dysfunction in long‐COVID: a hypothesis. ACS Chem Neurosci. 2021;12(4):573‐580. doi: 10.1021/acschemneuro.0c00793 33538586

[59] Song E , Zhang C , Israelow B , et al. Neuroinvasion of SARS‐CoV‐2 in human and mouse brain. J Exp Med. 2021;218(3):e20202135. doi: 10.1084/jem.20202135 33433624 PMC7808299

[60] Deleidi M , Isacson O . Viral and inflammatory triggers of neurodegenerative diseases. Sci Transl Med. 2012;4(121):121ps3. doi: 10.1126/scitranslmed.3003492 PMC3982831 22344685

[61] Hogestyn JM , Mock DJ , Mayer‐Proschel M . Contributions of neurotropic human herpesviruses herpes simplex virus 1 and human herpesvirus 6 to neurodegenerative disease pathology. Neural Regen Res. 2018;13(2):211‐221. doi: 10.4103/1673-5374.226380 29557362 PMC5879884

