# Supplementary material for: Comprehensive profiling of the human viral exposome in households containing an at‐risk child with mitochondrial disease during the 2020–2021 COVID‐19 pandemic
Source: Clin Transl Med. 2022 Nov 6;12(11):e1100. doi: 10.1002/ctm2.1100 (PMC9637669; doi:10.1002/ctm2.1100)
Supplement: Supplementary file 1 — Supporting Information [file CTM2-12-0-s001.docx]

**SUPPLEMENTARY DATA**

**Tables**

**
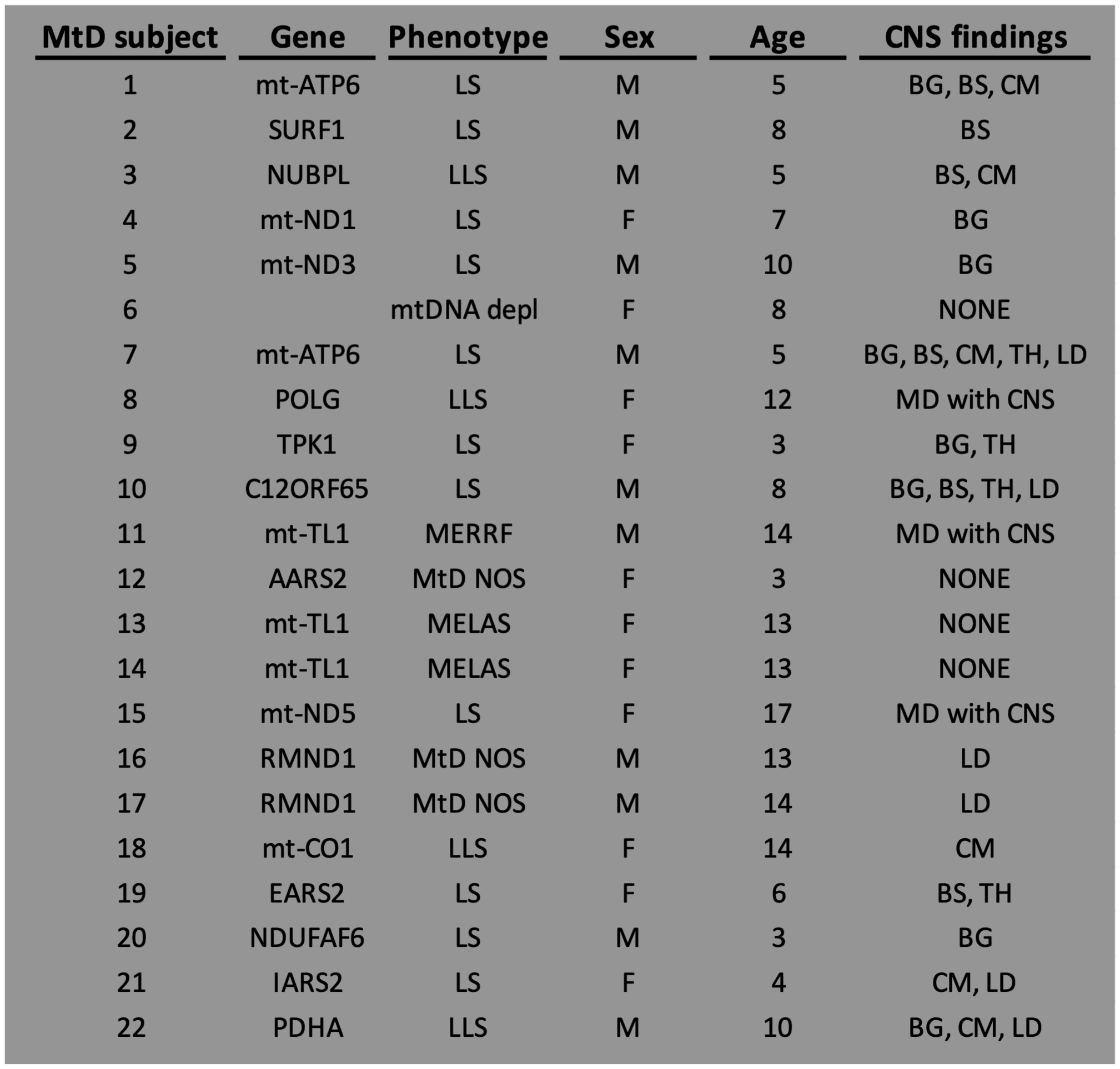
**

**Table S1: Clinical characteristics of children with MtD.** MtD = mitochondrial disease, LS = Leigh Syndrome, LLS = Leigh-like syndrome, mtDNA depl = mitochondrial depletion syndrome, MERRF = myoclonic epilepsy with ragged-red fibers, MELAS = mitochondrial encephalomyopathy, lactic acidosis, and stroke-like episodes, MtD NOS = mitochondrial disease not otherwise specified, CNS = central nervous system, BG = basal ganglia, BS = brain stem, CM = cerebellum, CX = cerebral cortex, WM = white matter, TH = thalamus.

**Table S2: Characteristics of various viruses identified by VirScan.** Each virus was denoted by other names, route of transmission, seasonality of infection, and symptoms.

**Figures**

**Figure S1: Risk factors for adverse outcomes during viral infections in children with MtD.** Medical records and structured interviews were conducted to identify risk factors for adverse outcomes during viral infections in children with MtD (N=22). POTS = Postural orthostatic tachycardia syndrome.

**Figure S2: Validation of Neoteryx capillary blood sampling platform.** Serum and capillary blood samples were collected from 5 healthy individuals for comparison. Both sets of samples were analyzed by VirScan and AVARDA. A) Linear regression of peptide counts for serum and Neoteryx samples. B) Identification of viral infections for Neoteryx and serum samples. Kappa = Cohen’s kappa statistic.

**Figure S3: IPMDS Domain 1 scores for hospitalized children with MtD.** Children with MtD who treated at home versus hospitalized for their illness were categorized and plotted for IPMDS score. Each point represents a child with MtD. **P < 0.01 Mann-Whitney Test.
